# Tetraploidy accelerates adaptation under drug selection in a fungal pathogen

**DOI:** 10.3389/ffunb.2022.984377

**Published:** 2022-11-16

**Authors:** Ognenka Avramovska, Amanda C. Smith, Emily Rego, Meleah A. Hickman

**Affiliations:** ^1^ Department of Biology, Emory University, Atlanta, GA, United States; ^2^ Department of Genetics, Harvard Medical School, Boston, MA, United States; ^3^ Division of Viral Disease, CDC Foundation, Atlanta, GA, United States

**Keywords:** *Candida albicans*, evolution, antifungals, ploidy, drug-resistance, fungal pathogen

## Abstract

Baseline ploidy significantly impacts evolutionary trajectories and, specifically, tetraploidy is associated with higher rates of adaptation relative to haploidy and diploidy. While the majority of experimental evolution studies investigating ploidy use the budding yeast *Saccharomyces cerivisiae*, the fungal pathogen *Candida albicans* is a powerful system to investigate ploidy dynamics, particularly in the context of acquiring antifungal drug resistance. *C. albicans* laboratory and clinical strains are predominantly diploid, but have been isolated as haploid and polyploid. Here, we evolved diploid and tetraploid *C. albicans* for ~60 days in the antifungal drug caspofungin. Tetraploid-evolved lines adapted faster than diploid-evolved lines and reached higher levels of caspofungin resistance. While diploid-evolved lines generally maintained their initial genome size, tetraploid-evolved lines rapidly underwent genome-size reductions and did so prior to caspofungin adaptation. While clinical resistance was largely due to mutations in *FKS1*, these mutations were caused by substitutions in diploid, and indels in tetraploid isolates. Furthermore, fitness costs in the absence of drug selection were significantly less in tetraploid-evolved lines compared to the diploid-evolved lines. Taken together, this work supports a model of adaptation in which the tetraploid state is transient but its ability to rapidly transition ploidy states improves adaptive outcomes and may drive drug resistance in fungal pathogens.

## Introduction

Variation in ploidy (i.e. the number of chromosome sets in a genome) occurs widely across the tree of life with differences between species, within species, and even in cell types of a single organism (Otto, 2007; Otto and Gerstein, 2008; De Peer et al., 2017). The ploidy spectrum ranges from haploidy to diploidy to polyploidy, each state holds unique evolutionary benefits and drawbacks based on ploidy specific mutation rates and effect sizes (Mable and Otto, 2001; Otto and Gerstein, 2008; Gerstein, 2013; Selmecki et al., 2015; Sharp et al., 2018; Gerstein and Sharp, 2021). However, an exhaustive study comparing more than 12,000 growth curves of haploid and diploid *Saccharomyces* in 33 conditions found no global evolutionary advantage to haploidy or diploidy, which suggests that the effects of ploidy on adaptation are more intricate than initially hypothesized (Zörgö et al., 2013). Compared to haploids and diploids, polyploids generally exhibit much higher levels of genome instability and ability to generate mutations and genomic rearrangements (Mayer and Aguilera, 1990; Storchová et al., 2006; Hickman et al., 2015; Avramovska and Hickman, 2019). In clonal populations, high mutation rates are advantageous if the limiting factor to adaptation is generating mutations. However, if beneficial mutations are recessive, they will take longer to be unmasked in polyploids (Otto, 2007; De Peer et al., 2017). To unmask beneficial or purge deleterious mutations, polyploids can undergo ploidy reductions. In raffinose-limiting conditions during experimental evolution, tetraploid yeast undergo large-scale ploidy reductions (to diploidy) by the experimental endpoint and also adapt more quickly compared to haploids and diploids (Selmecki et al., 2009; Sionov et al., 2010). In addition to yeast, polyploidy has been linked to increased adaptability in human hepatocyte cells experiencing DNA-damage (Duncan, 2013) and in polyploid root tips of *Arabidopsis thaliana* undergoing salt stress (Chao et al., 2013). Taken together, polyploidy and the ability to undergo ploidy transitions impacts evolutionary trajectories across organisms.

Polyploidy is frequently observed in human pathogenic fungi such as *Cryptococcus neoformans* (Okagaki and Nielsen, 2012; Gerstein et al., 2015) and *Candida albicans* (Magee and Magee, 2000; Bennett and Johnson, 2003; Forche et al., 2008). Titan cells of the basidiomycete *C. neoforms* arise through endoreplication and range from 4C – 32C genome content (Zaragoza and Nielsen, 2013). These unstable polyploids produce daughter progeny of various karyotypes, some of which are more stress-tolerant and resistant to antifungals (Okagaki and Nielsen, 2012; Gerstein et al., 2015). Tetraploids of the ascomycete *C. albicans* arise through cell-cell fusion of diploid cells with opposite mating types (Magee and Magee, 2000; Bennett and Johnson, 2003; Forche et al., 2008). Tetraploid *C. albicans* cells have elevated rates of loss-of-heterozygosity (Hickman et al., 2015; Avramovska and Hickman, 2019) and undergo random chromosome loss to generate diverse near-diploid progeny that carry aneuploid chromosomes (Hickman et al., 2015; Hirakawa et al., 2017). Aneuploidy and its associated increase in copy number of resistance-associated genes, such as *TAC* and *ERG*, is a mechanism by which resistance to fluconazole, a commonly used antifungal, arises in clinical and laboratory isolates (Selmecki et al., 2009; Sionov et al., 2010). Therefore, tetraploids’ propensity for chromosome loss may be an important mechanism driving adaptation in under antifungal-drug selective pressure in asexual eukaryotic organisms.

*In-vitro* experimental evolution is a powerful tool to answer key hypotheses regarding ploidy drive (Gerstein et al., 2017) mechanisms of drug resistance (Cowen et al., 2000) and fitness effects of these mutations (Cowen et al., 2001; Selmecki et al., 2009; Popp et al., 2017) in the fungal pathogen, *Candida albicans*. Through this approach, mutations conferring drug resistance to the azole, fluconazole (Cowen et al., 2000; Selmecki et al., 2006; Selmecki et al., 2009) in *C. albicans* have been identified. For the isolates evolved in fluconazole, resistance is associated with overexpression of the drug pumps *CDR1/2*, drug target *ERG11*, and transcriptional regulator, *MDR1* (Cowen et al., 2000). The overexpression of these genes is linked to aneuploidy of isochromosome 5L (Selmecki et al., 2006; Selmecki et al., 2009). Studies assessing the fitness effects of fluconazole resistance mutations yield variable results. *C. albicans* fitness costs determined through direct competition assays of resistant and sensitive isolates depended on the specific type of mutation conferring resistance. Notably, there is no association between observed fitness costs *in-vivo* and *in-vitro* (Popp et al., 2017). In another set of studies, fitness costs to fluconazole resistance that arose during experimental evolution in *C. albicans* were eliminated by further adaptation (Cowen et al., 2001) and the aneuploidy driving fluconazole resistance (i5L) was not associated with a fitness cost in the absence of selection (Selmecki et al., 2009).

*In-vitro* experimental evolution approaches have identified key resistance mechanisms to two separate types of drug classes, the azoles and the polyenes, though this approach has yet to be applied to echinocandin drugs and *Candida albicans*. Unlike azoles, which target the cell membrane and inhibit the growth of cells (fungistatic) (Revie et al., 2018), echinocandins such as caspofungin are fungicidal, and able to effectively kill yeast cells through activating apoptotic pathways (Hao et al., 2013). Echinocandins elicit cell wall stress through inhibition of β 1-3 glucan synthase, an enzyme required for production of an inner cell wall polysaccharide β 1-3 glucan. Through sequencing of clinically resistant *Candida albicans*, mutations in the hotspot region of the drug target of caspofungin, *FKS1* have emerged as causes of clinical resistance (Balashov et al., 2006; Garcia-Effron et al., 2009; Spettel et al., 2019). In addition to point-mutations, aneuploidy of chromosome 5 and 2 (Sah et al., 2021) and remodeling of the fungal cell wall through increased chitin synthesis (Lee et al., 2012; De Cesare et al., 2022) are mechanisms linked to echinocandin tolerance. However, fitness costs associated with caspofungin resistance are inconclusive. In an invertebrate model, *Galleria mellonella*, larvae infected with caspofungin-resistant *Candida glabrata* have the same survival and larvae infected with drug-sensitive yeast (Borghi et al., 2013), supporting the idea that there is no fitness cost to caspofungin resistance. Yet, a *Drosophila melanogaster* larvae infection model shows larvae infected with caspofungin resistant *Candida albicans* isolates survive longer than WT and did incur a fitness cost (Ben-Ami et al., 2011). Considering these contrasting results, additional studies are necessary to disentangle the fitness costs of echinocandin resistance.

There are a limited number of studies that have investigated how tetraploidy contributes to the emergence of fluconazole drug-resistance in fungal pathogens (Okagaki et al., 2010; Harrison et al., 2014) and a minimal number of studies that have examined the role of ploidy in caspofungin drug-resistance. We, and others, have previously shown that tetraploids have both higher baseline and drug-induced genome instability compared to diploids (Mayer and Aguilera, 1990; Storchova and Kuffer, 2008; Hickman et al., 2015; Avramovska and Hickman, 2019). Additionally, in nutrient-limiting conditions, tetraploid yeast adapted faster than both haploids and diploids. Thus, we hypothesize that tetraploidy facilitates rapid adaptation to antifungal drugs. We evolved ~70 diploid and tetraploid lines in 0.25µg/ml caspofungin for 60 days and tracked adaptation and genome size throughout evolution. We found that tetraploid-evolved lines adapt faster and reach higher resistance levels than diploid-evolved lines. Furthermore, large-scale genome-size reductions occurred prior to adaptation, though only in tetraploid-evolved lines. To understand the fitness costs of caspofungin adaptation, we measured growth rates in the absence of drug and found ploidy-specific differences, with tetraploids incurring half the fitness costs of diploids. Interestingly, we did not detect differences between the growth rates of caspofungin-susceptible and caspofungin-resistant lines, suggesting there was not a fitness cost to caspofungin resistance. In conclusion, we demonstrate that tetraploidy is transient and can facilitate adaptation in asexual organisms.

## Materials and methods

### Yeast strains and media

Stains used in this study are listed in **Table S1**. All evolved lines were archived in 50% glycerol and stored at −80°C in 96-well block format. Lines were maintained on YPD (1% yeast extract, 2% bactopeptone, 2% glucose, 1.5% agar, 0.004% adenine, 0.008% uridine) or casitone (0.9% bacto-casitone, 0.5% yeast extract, 1% sodium citrate, 2% glucose, 1% agar) media at 30°C. Liquid yeast cultures were grown in casitone (0.9% bacto-casitone, 0.5% yeast extract, 1% sodium citrate, 2% glucose). A stock solution of caspofungin of 1mg/ml was made from powder (Sigma-Aldrich CAS#179463-17-3) and suspended into ddH_2_0.

### Experimental evolution

Diploid strain MH84 and tetraploid strain MH128 were initially struck onto YPD agar plates to obtain single colonies. 72 diploid single colonies and 72 tetraploid single colonies were inoculated into YPD and cultured for 24 hrs. The following day, cultures were normalized to 0.05 OD, and 100uL of the diluted cells were added to 900uL of casitone supplemented with 0.25µg/mL caspofungin and 100µg/mL streptomycin (to prevent bacterial growth) in a 96-well block. Culture blocks were covered with BreathEasy tape, placed in plastic lidded containers containing damp paper towels to maintain humidity and incubated at 30°C. Every 7 days, 100uL was removed for archival glycerol stocks. The remaining cultures were pelleted by centrifugation (2 min at 1500 RPM), media removed and pellets were resuspended in 1mL fresh casitone media supplemented with 0.25µg/mL caspofungin and 100µg/mL streptomycin. After the experimental endpoint, all replicate lines were assessed for contamination and subsequently, seven diploid replicate lines were removed from the analysis.

### Relative caspofungin growth

Relative caspofungin growth (RCG) was measured every 7 days by spotting 5uL of evolved cultures onto casitone (no-drug) and casitone containing 0.25µg/mL caspofungin (+drug) agar plates. Plates were incubated at 30°C for 24 hrs and subsequently photographed. Photographs were analyzed to determine the pixel cell area for each spot using Colonyzer imaging software (Lawless et al., 2010). Relative caspofungin growth was calculated by dividing the pixel area of each spot on the +drug plates by the pixel area into the corresponding no-drug plate. Ratios were capped at 1.0, indicating no growth difference in the presence or absence of caspofungin.

### Drug susceptibility

E-test: Minimum inhibitory concentrations (MIC) were measured as previous described (Avramovska and Hickman, 2019). Briefly, 10µL of glycerol stock was inoculated into 2 mL YPD supplemented with 100µg/mL ampicillin and grown with shaking at 30°C for 24hrs. Cultures were normalized to 0.1 OD with ddH_2_0. 200uL was spread onto casitone agar (1%) plates and left to dry for 15 min at 30°C. Standardized caspofungin E-test strips (gradient 0.002 µg/mL – 32 µg/mL; Biomeureix) were added to the middle of the plates and incubated at 30° C for 24 hrs and subsequently photographed.

Microbroth Dilution Assay: Microbroth dilution assays were performed as in the CLSI M27-A guidelines with the following modifications. Evolved lines were inoculated into casitone media supplemented with 100µg/mL ampicillin and incubated at 30°C with shaking for 48 hrs. Cultures were normalized to 1 OD and diluted to yield approximately 1x10^3^ cells/mL. 100uL of the diluted cells were added to 100uL of casitone containing a gradient of caspofungin concentrations (no-drug, 0.03125µg/mL caspofungin - 4µg/mL). Blocks were covered with BreatheEasy tape and incubated at 30° C for 24 hrs. OD_600_ was measured on a plate reader (BioTekGen5). The drug concentration in which the fraction of growth (relative to no-drug) was below 0.5 was considered the minimum inhibitory concentration.

### Growth rates

Growth rates were determined similar to (Hickman et al., 2015) with some modification. Evolved lines were inoculated from 10µL of glycerol stock into 490uL of YPD with 100 µg/mL ampicillin and grown for 24hrs with shaking at 30°C to recover cells. Following 24 hrs growth, 1:200 dilution was performed into fresh YPD media and OD_600_ was measured every 15 min with shaking using BioTek5 growth reader. Growth rate were determined using R-script which uses the spline function to fit a line of best-fit for each growth curve (Gerstein et al., 2012).

### Flow cytometry

Flow cytometry analysis was performed as previously published (Hickman et al., 2015; Avramovska and Hickman, 2019). Initially, 200uL of midlog-phase cells were harvested, washed with distilled water, and resuspended in 20uL of 50:50 TE (50 mM Tris pH 8 and 50 mM EDTA). Cells were fixed with 95% ethanol and incubated at 4°C overnight. Following ethanol fixation, cells were washed twice with 50:50 TE, resuspended in 50µL of 1 mg/ml RNAse A and incubated at 37°C for 1-3hrs. Cells were collected, resuspended in 50µL of 5 mg/ml Proteinase K, and incubated at 37°C for 30min. Cells were subsequently washed once with 50:50 TE, resuspended in 50µL SybrGreen (1:100 of dilution in 50:50 TE, Lonza, CAT#12001-798) and incubated overnight at room temperature. Cells were collected *via* centrifugation and resuspended in 150µL 50:50 TE, briefly sonicated, and run on a LSRII machine with laboratory diploid (MH1) and tetraploid (MH2) strains serving as calibration and internal controls. To estimate the average G1 peak (FITC-A or BB515 intensity), the multi-Gaussian cell cycle model was used (FloJoV10). Each G1 peaks measurement represents the mean genome size of at least 10,000 events per evolved-line.

### Whole-genome sequencing

Genomic DNA was isolated with phenol chloroform as described previously (Selmecki et al., 2006). Whole genome sequencing was performed through the Microbial Genome Sequencing Center using a single library preparation method based on the Illumina Nextera kit. Libraries were sequencing using paired end (2 x 150 bp) reads on the NextSeq 550 platform. Adaptor sequences and low-quality reads were trimmed using Trimmomatic (v0.39 LEADING:3 TRAILING: 3 SLIDINGWINDOW: 4:15 MINLEN: 36 TOPHRED33) (Bolger et. al,2014. All reads were mapped to the *C. albicans* reference genome using the Burrows-Wheeler Aligner MEM (BWA v0.1.19) algorithm to align the sequencing reads to the reference genome followed by Samtools (v0.1.19) to sort, mark duplicates, and create a BAM file. Identification of aneuploidy, CNVs, and LOH were conducted using whole genome sequencing data and the Yeast Mapping Analysis Pipeline (YMAP). BAM files were uploaded to YMAP and plotted using the Candida albicans reference genome (A21-s02smo8-r09) with corrections for chromosome end bias and GC content (Abbey et. al, 2014)

### Statistical analysis

Non-parametric t-tests (Kruskall-Wallis with Dunn’s *post-hoc*) were used when comparing all diploid timepoints to all tetraploid timepoints (all-by-all). Unpaired Mann-Whitney U-test were used when determining significance between diploids and tetraploids of the same timepoint. GraphPad PRISM 9 software was used for these calculations.

## Results

### Tetraploids evolve faster and attain higher antifungal resistance than diploids

**Figure 1:**
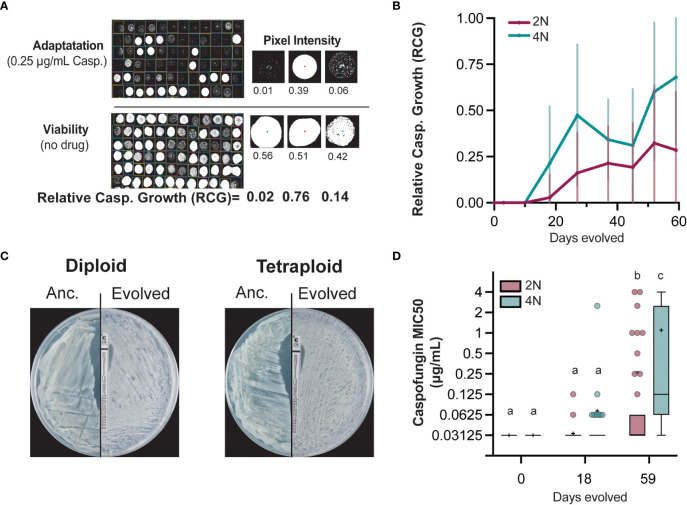
Diploid and tetraploid *C. albicans* evolution under caspofungin selection.**(A)** Relative caspofungin growth (RCG) was determined periodically over the course of experimental evolution. Replicate lines were spotted on no-drug and 0.25μg/mL caspofungin plates and photographed after 24 hrs. Pixel intensity was measured and the ratio of caspofungin growth to total viable growth calculated. Representative images with calculated RCG are tetraploid lines 68, 69, and 70 from Day45. **(B)** Average RCG for diploid (maroon; n=65) and tetraploid (teal; n=72) replicate lines on days 0, 3, 10, 18, 27, 37, 45, 52, 59. Error bars represent +/- 1SD. **(C)** Representative caspofungin MIC e-tests of ancestral and day 59 evolved diploid (line 64) and tetraploid (line 35). **(D)** Caspofungin liquid MIC of diploid- and tetraploid-evolved lines for day 0, day 18, and day 59. Data is displayed as Tukey- box-and-whisker plots. The mean is indicated by (+) and circles indicate outliers. Statistical differences among ploidy group and timepoints were tested using Kruskal-Wallis test with Dunn’s *post hoc* multiple-comparison testing. Groups that share letters are not significantly different, whereas those with differing letters are statistically different.

To test the hypothesis that tetraploid *Candida albicans* will adapt faster to caspofungin, we quantified the adaptive evolution of 65-diploid and 72-tetraploid lines in the antifungal drug caspofungin. We evolved the replicate lines in 0.25µg/mL caspofungin, a concentration that reduces viability by 99% (Avramovska and Hickman, 2019), for 59 days. Given the fungicidal nature of caspofungin, replicate lines were not subjected to bottlenecks, instead selective media was replenished every week. Throughout the course of experimental evolution, we simultaneously spotted replicate lines onto selective (+drug) and non-selective (no drug) media and photographed 24 hrs later for image analysis (**Figure 1A**). For each replicate line, we calculated the relative caspofungin growth (RCG) by dividing the area of growth on selective media by the area of growth on non-selective media for days 0, 3, 10, 18, 27, 37, 45, 52 and 59 (**Figures 1A, B**, **S31**). Detectable RCG changes were initially observed on day 18, with an average RCG of 0.02 and 0.21 for diploid- and tetraploid-evolved lines, respectively. Day 18 tetraploid-evolved RCG was largely driven by 19 evolved lines with values greater than 0.5 (**Figure S1B**). In contrast, only a single diploid-evolved line had an RCG greater than 0.5 at this timepoint. Throughout the evolution, tetraploid-evolved lines had significantly higher average RCG than diploid-evolved lines (**Figure 1B**). By day 59, tetraploid-evolved RCG was 0.68 compared to the 0.28 diploid-evolved RCG. This data supports the hypothesis that tetraploids not only adapt more rapidly than diploids, they improve by significant margins.

While RCG captures caspofungin adaptation over the course of experimental evolution, it may not necessarily reflect caspofungin resistance, a clinical term defined as a minimum inhibitory concentration (MIC) of at least 1µg/mL (Santos et al., 2014). MIC is measured either by growth on agar plates with a drug concentration gradient (i.e. e-tests, **Figure 1C**) or in liquid culture as a microbroth dilution assay, which was also implemented in this study (**Figure 1D**). We determined the MIC for all replicate lines on days 0, 18, and 59 (**Figure 1D**). The ancestral MIC was 0.03125 µg/ml for both ploidy states despite ancestral and by day 18, the diploid-evolved MIC had not changed from the ancestral, whereas tetraploid-evolved MIC had increased to 0.07 µg/ml. At the final timepoint, day 59, tetraploid-evolved MIC was still significantly higher (1.0µg/ml) than diploid-evolved MIC (0.4µg/ml), a pattern consistent with RCG values (Kruskal-Wallis, p<0.0001, **Table S2**).

We assessed the relationship between MIC and RCG and found that evolved-lines with MIC values ≥ 0.25µg/ml caspofungin have RCG values 0.71 or greater. However, this relationship breaks down for evolved lines with MICs less than 0.25µg/ml (**Figure S2**). Therefore, we measured changes in MIC, rather than RCG in subsequent analyses. By day 18, 13% (9/70) of tetraploid-evolved lines, and 3% (2/65) diploid-evolved lines had increased MIC by at least two-fold (**Figures S2A, B**). At the end of the evolution experiment, nearly all the tetraploid-evolved lines (70/72) had increased MIC, whereas only half of the diploid-evolved lines (28/65) had improved (**Figures S2C, D**). Interestingly, 3% (3/65) of diploid-evolved lines and nearly 40% (28/72) of tetraploid-evolved lines had MIC values that ten-fold or higher compared to the selective pressure drug concentration they were evolved in (**Figures S2C, D**). Regardless of how growth in caspofungin was quantified, we found that tetraploids evolved faster than diploids and more frequently attained drug-resistant phenotypes.

### Large scale genome size reductions occur prior to adaptation

**Figure 2:**
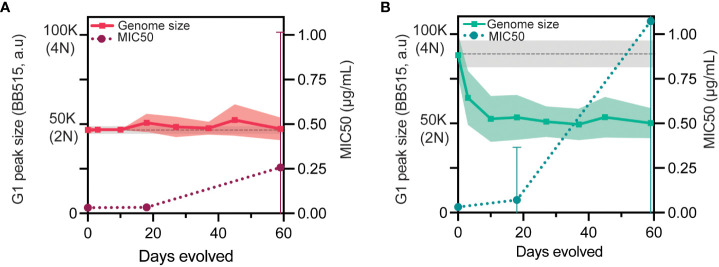
Large scale genome size reductions occur prior to adaptation. **(A)** Diploid genome size measured throughout experimental evolution with the mean (solid line) and +/- 1SD (filled-pink) of G1 peaks plotted in a.u (arbitrary units) on the left y-axis. The gray dashed line indicates the mean of the ancestral diploid controls with +/-1SD (filled-gray, n=72). Average MIC50 (maroon dotted-line) is plotted on the right y-axis with +/-1SD. **(B)** Tetraploid genome size measured throughout experimental evolution with the mean (solid line) and +/- 1SD (filled-green) of G1 peaks plotted in a.u (arbitrary units) on the left y-axis. The gray dashed line indicates the mean of the ancestral diploid control with +/-1SD (filled-gray, n=64). Average MIC50 (teal dotted-line) is plotted on the right y-axis with +/-1SD.

*C. albicans* tetraploid genomes are intrinsically unstable and frequently undergo chromosome loss to return to a diploid, or near-diploid state over time (Bennett and Johnson, 2003; Forche et al., 2008; Hickman et al., 2015; Gerstein et al., 2017). We have previously shown that short-term exposure to caspofungin rapidly induces chromosome loss in tetraploids (Avramovska and Hickman, 2019) and thus, we next investigated whether there was any relationship between changes in genome size and adaptation to caspofungin. Throughout the experimental evolution, we measured the genome size for the diploid- and tetraploid-evolved lines (**Figures 2A, B**, left y-axis and **Figure S3**) and observed that tetraploids reduced extensively in genome size within the first 10 days. This contrasts with diploids, which largely maintained their initial genome size throughout the evolution. It is at this timepoint that diploid-evolved and tetraploid-evolved lines have approximately the same genome-size, suggesting that tetraploid-evolved lines have reached a near diploid state (**Figure 2B**, **Figure S3**). We then plotted the average MIC for the replicate lines (**Figures 2A, B**, right y-axis) and compared whether the increase in average MIC coincided with overall changes in genome size. As a control, we passaged 24 tetraploid replicate lines for 28 days in no-drug (YPD, **Figure S5**). While nearly all tetraploids lost genome content to reach a diploid genome, there was no change in the MIC. For the tetraploid replicate lines, the average G1 peak genome sizes were approximately triploid by day 3, and approximately diploid by day 10. However, these genome size reductions did not coincide with increases in average MIC, as only 9/70 lines showed improvement in MIC values. From this data, we conclude that tetraploids rapidly reduce in genome size, however this occurs prior to caspofungin adaptation.

### Caspofungin resistant isolates have chromosomal copy-number variation

**Figure 3:**
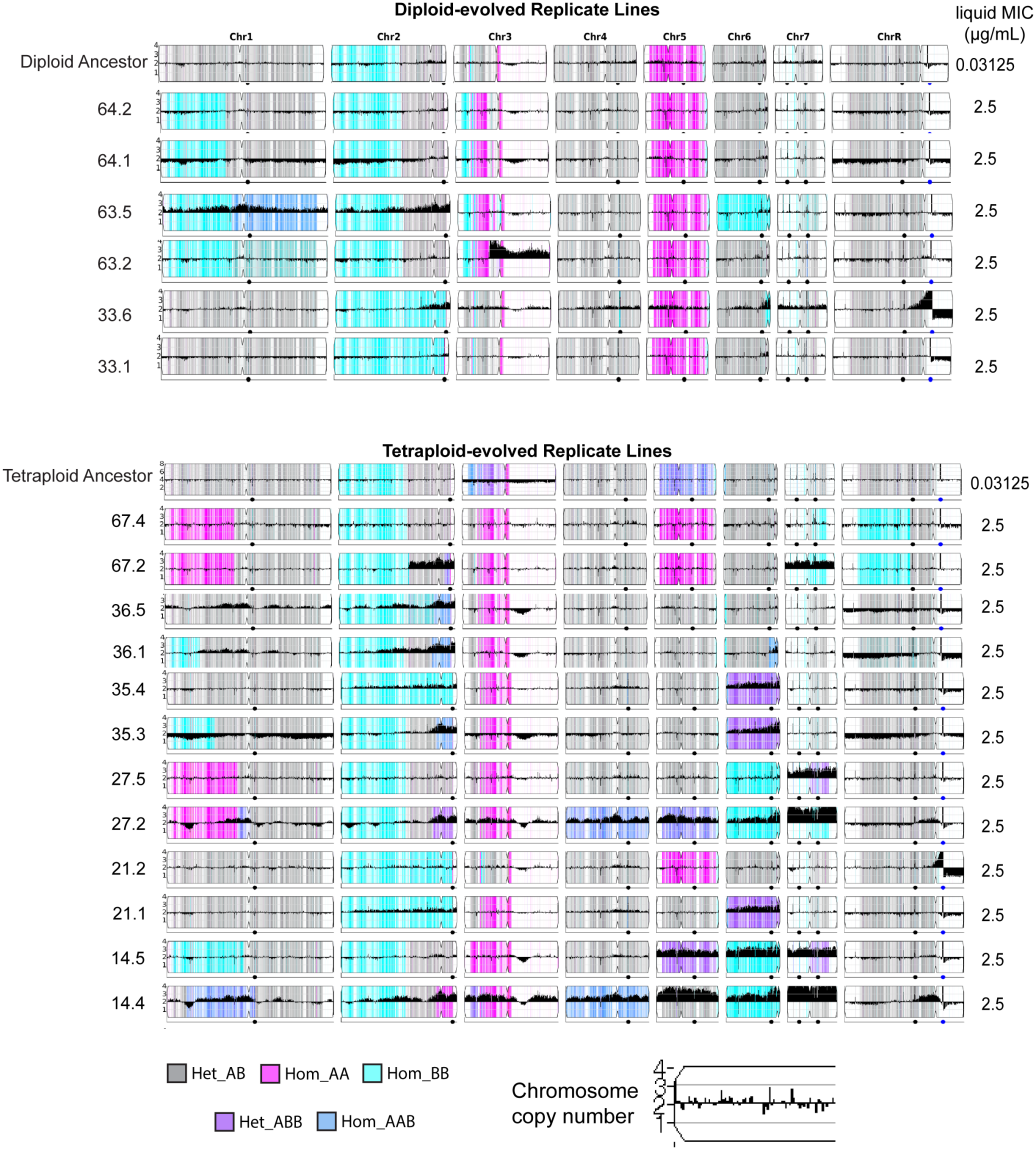
Genome-wide changes following evolution in caspofungin. YMAPs of *C.albicans* diploid (top) and tetraploid-evolved (bottom) isolates on day59 of evolution in caspofungin. Reference diploid and reference tetraploid are displayed at the top of each respective sections. Two single colonies (isolates) were sequenced is per replicate line. Chromosome copy number is indicated on each chromosome and chromosomal color indicates allelic ratio (gray = heterozygous, cyan/pink = homozygous, darker blue/purple = heterozygous with >2 alleles). MIC on day59 is shown on the right of each YMAP.

Recently, trisomy of chromosome 2 (Yang et al., 2019) and copy number variation of chromosome 5 (monosomy and iso-5R) have shown improved caspofungin tolerance (Yang et al., 2017; Sah et al., 2021). To identify genome changes that occurred during the experimental evolution of clinically resistant replicate lines (MIC = 2.5 μg/mL), six diploid-evolved isolates and twelve tetraploid-evolved isolates were whole genome sequenced. We sequenced two single colonies (isolates) per replicate line to capture potential variation within the lineages. To identify specific aneuploidies and tracts of loss-of-heterozygosity, all genomes were visualized using YMAP (**Figure 3**). Out of the six sequenced diploid-evolved isolates, none contained whole-chromosome aneuploidy. However, two (lines 33.1 and 63.5) had copy-number variation of the right-arm of chromosome 2, By contrast, of the 12 tetraploid-evolved lines that were sequenced, all had returned to approximately diploid genomes, though 92% (11/12) carried an aneuploidy of at least one chromosome (**Figure 3**). The most common copy-number variations were of the right arm of chromosome 2 and whole-chromosome duplication of chromosome 6, both which occurred in 50% of isolates. Interestingly, all isolates carrying aneuploidy of Chromosome 6 maintained 2 copies of the B-homolog. Chromosome 7 trisomy and tetrasomy were also observed in 42% (5/12) isolates. In addition to copy-number variation, reassortment of alleles through chromosome loss or loss-of-heterozygosity was also detected in the evolved isolates. In diploids these events were detected on chromosome 1, 2, 6 and 7; however, in tetraploids, these events were detected across all eight chromosomes (**Figure 3**).

**Figure 4:**
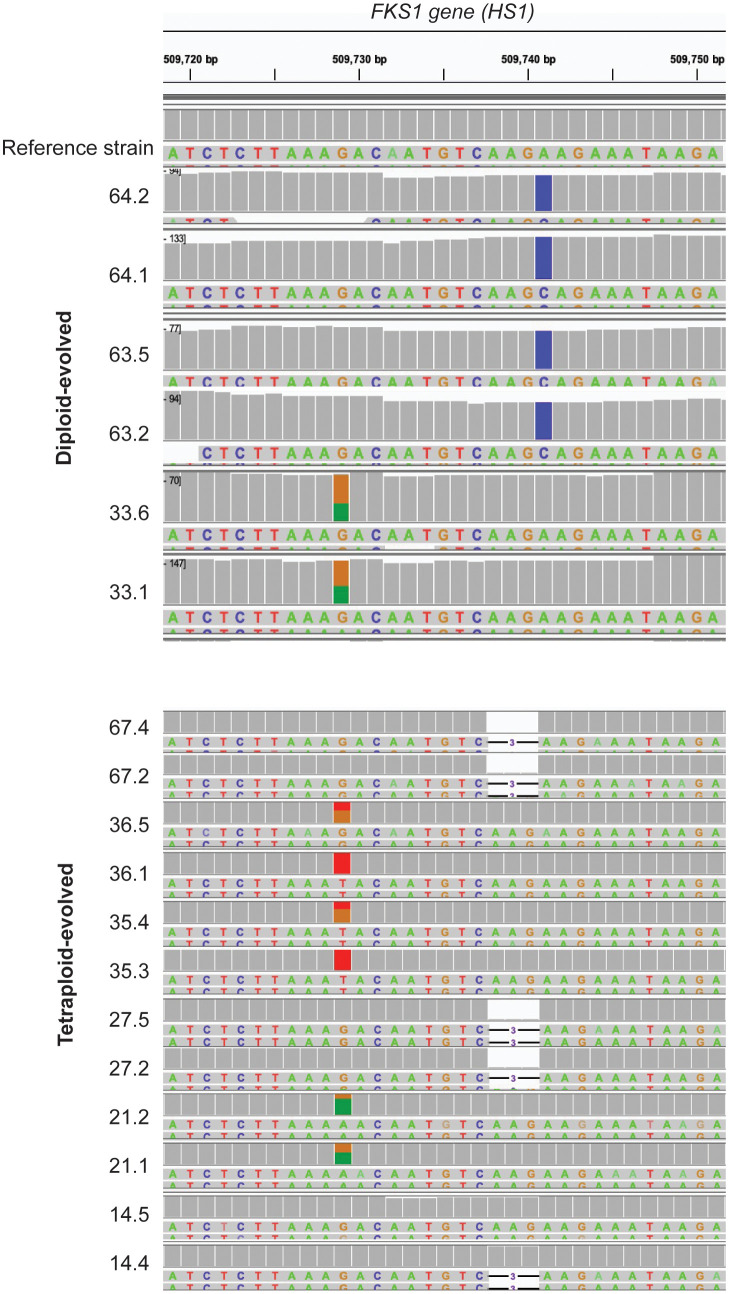
*FKS1* mutations differ between diploid- and tetraploid-evolved isolates. SNPs in the HS1 region of FKS1 if *C.albicans* diploid (top) and tetraploid-evolved (bottom) isolates on day59 of evolution in caspofungin. Each colored nucleotide represents a SNP in the region. A fully colored box represents a homozygous SNP, while partially colored boxes represent heterozygous snips.

While large-scale copy number variations and loss-of-heterozygosity events were observed across resistant isolates (**Figure 4**), we also wanted to identify SNPs that may contribute to resistance. We scanned the entirety of *FKS1* for the diploid and tetraploid isolates and identified mutations in hotspot region 1 (HS1) for 6/6 diploids and 11/12 tetraploid isolates (**Figure 4**). In diploid-evolved lines, all isolates from replicate lines 63 and 64 were homozygous at F641C while both isolates from replicate line 33 were heterozygous at S654F mutation. Similarly, six tetraploid isolates had mutations at position S645, with two isolates heterozygous at S654F (21.1 and 21.2), two heterozygous at S645Y (36.5 and 35.4) and two homozygous at S645Y (36.1 and 35.3). While the vast majority of previously identified *FKS1* mutations in *Candida albicans* are substitutions, nearly half of the tetraploid-evolved lines (5/12) had a 3 base pair deletion at the first amino acid of the hotspot (F641). While this mutation has not yet been observed in *Candida albicans*, it has been associated with multi-echinocandin resistance in *Candida kefyr, Candida glabrata* and *Candida auris* (Staab et. al, 2014; Carolus et al., 2021). One replicate line (14.5) reached clinical resistance and did not have mutations in *FKS1*. Our findings support the hypothesis that mutational spectrum differs based on ploidy and in plays a role in caspofungin resistance. (Staab et al., 2014)

### Tetraploids incur lower fitness costs in the absence of caspofungin selection

**Figure 5:**
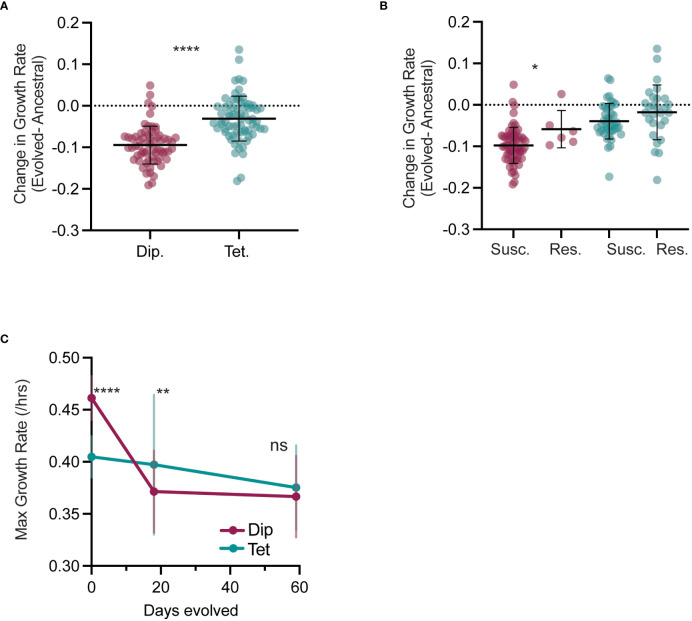
Tetraploids have lower fitness costs in the absence of caspofungin.**(A)** The change in growth rate (D59-D0) for each diploid (maroon) and tetraploid (teal) evolved line is plotted, with the black horizontal lines representing the mean, and vertical lines representing the SD. Each symbol represents the average of two replicate experiments. Statistical comparison between diploid and tetraploid is represented by Mann-Whitney U-test (****p < 0.0001). **(B)** The change in growth rate (D59-D0) for each sensitive and resistant diploid (maroon) and tetraploid (teal) evolved line is plotted, with the black horizontal lines representing the mean, and vertical lines representing the SD. Each symbol represents the average of two replicate experiments. Statistical comparison is between sensitive and resistant populations of the same ploidy and is represented by Mann-Whitney U-test (**p<0.01). **(C)** Mean and SD growth rates of diploid (maroon) and tetraploid (teal) evolved lines at day 0, 18 and 59. Statistical comparison is between average diploid and tetraploid growth rate form the same day and is represented by Mann-Whitney U-test (****p < 0.0001, **p < 0.01).

During caspofungin experimental evolution, we found that total viable growth was highly variable, with some lines growing robustly and others barely viable (**Figure 1A**, no drug), likely from potentially deleterious mutation accumulation induced by caspofungin (Avramovska and Hickman, 2019). To test whether caspofungin evolution resulted in reduced fitness in the absence of drug, we measured the difference between growth rates of the evolved lines (day 59) and ancestral lines (day 0) (**Figure S5A**). We hypothesized if the ancestral growth rate is greater than evolved growth rate in no-drug (ΔGrowth Rate < 0), then evolution under caspofungin selective pressure generated deleterious mutations leading to a decrease in fitness in the absence of selection. While the ancestral growth rates were greater in both diploid and tetraploid-evolved lines, suggesting a fitness cost to caspofungin exposure, the diploid cost (ΔGR = -0.09) was 3-times that of tetraploid (ΔGR= -0.03) (**Figure 5A**). However, the initial growth rates of tetraploid lines were lower than diploid lines (p < 0.0001 Mann-Whitney U-test, **Figure S5A**) and there was a significant negative relationship between initial and evolved growth rates (**Figure S5B**, R^2^ = 0.51, p < 0.0001); lines with higher initial growth rates had greater reductions in growth rates following caspofungin evolution.

To test if the reductions in no-drug growth rates were a trade-off with caspofungin resistance, we compared growth rates of evolved lines with an MIC greater than or equal to 1.0 µg/ml caspofungin (‘resistant’) to the evolved lines with an MIC less than 1µg/ml (‘susceptible’) in the absence of drug (**Figure 5B**). For diploids, the resistant evolved lines had slightly smaller fitness costs compared to the susceptible lines, while for tetraploids, there was no difference in no-drug growth rates between resistant and susceptible evolved lines. Thus, reductions in no-drug growth rates were not due to a direct trade-off with resistance to caspofungin and rather, accumulation of deleterious caspofungin-induced mutations.

We next measured no-drug growth rates on day 18, a timepoint with minimal detectable caspofungin adaptation, yet significant genome-size changes (**Figures 5C**, **S5C, D**, **TS2**). In diploid-evolved lines, by day18 there was significant reduction in no-drug growth rates compared to day 0, but no further reduction in growth rates by day 59. (**Supplemental Table 2**). However, in tetraploid-evolved lines, day18 growth rates were comparable to day 0 and to day 59 despite massive reductions in genome size (TS2). Additionally, on day 18, the mean growth rates for diploid-evolved lines were significantly less than tetraploid-evolved lines (**Figure 5C**, p < 0.01), though by the experimental endpoint, there was no differences in the growth rates. From these results, we propose that tetraploidy buffers against the early fitness costs of drug-induced mutagenesis by purging chromosomes with deleterious mutations. Nonetheless, once tetraploid genomes have reduced to diploid or near-diploid states, they accumulate mutations which result in fitness costs in the absence of selection.

## Discussion

In this study, we evolved diploid and tetraploid *C. albicans* under selection to the antifungal drug caspofungin to compare how ploidy impacts evolutionary trajectories, and found tetraploids adapted more rapidly and achieved higher levels of drug resistance compared to diploids. Early in the experiment (day 18) tetraploid-evolved MIC was twice that of ancestral, yet diploid-evolved lines showed no changes in MIC (**Figure 1**). By the end of the evolution tetraploid-evolved lines improved their MIC by 32-fold, compared to diploid-evolved lines that improved only by 2.5-fold. Our work demonstrates that tetraploidy can facilitate adaptation, a result that is consistent with other experimental evolution studies investigating the role of ploidy in adaptive processes. For example, *S. cerevisiae* tetraploids evolved under raffinose selection also adapted at a significantly faster rate than diploids (Selmecki et al., 2015). In part, accelerated adaptation may be due to elevated mutation rates observed in polyploids relative to diploids across yeast species (Mayer and Aguilera, 1990; Storchová et al., 2006; Hickman et al., 2015), leading to higher frequencies of beneficial mutations (Selmecki et al., 2015).

In addition to higher mutation rates, tetraploid cells also frequently undergo random chromosome loss and reduce in genome size under a diverse set of growth conditions *in vitro* and *in vivo* (Bennett and Johnson, 2003; Forche et al., 2008; Hickman et al., 2015; Gerstein et al., 2017; Avramovska and Hickman, 2019; Smith and Hickman, 2020). Interestingly, we found that many of the tetraploid-evolved lines rapidly reduced in genome size by day 10, a timepoint prior to when we first observed any detectable caspofungin adaptation (**Figure 2A**). Despite returning to diploid (or near-diploid) genome sizes, tetraploid-evolved lines still adapted more quickly and with larger increases in MIC than diploid-evolved lines. Non-meiotic ploidy reduction in *C. albicans* increases phenotypic variation amongst isolates derived from tetraploid cells (Hickman et al., 2015; Hirakawa et al., 2017). We propose that tetraploids are capable of accelerated adaptation because ploidy reduction generates derivatives whose chromosomes have been reassorted (**Figure 3**), thus carrying new combinations of alleles and derivatives that may contain chromosomal aneuploidy (Forche et al., 2008; Hickman et al., 2015; Hirakawa et al., 2017).

Aneuploidy is considered to be a ‘quick-fix’ that organisms use during adaptation by altering gene expression and protein abundance (Rancati et al., 2008; Pavelka et al., 2010; Yona et al., 2012). Specifically, chromosomal aneuploidy drives heat-tolerance in *Saccharomyces cerevisiae* (Yona et al., 2012), chemotherapy resistance in cancer cells (Duesberg et al., 2000; Gordon et al., 2012) and drug resistance in fungal pathogens (Selmecki et al., 2006; Selmecki et al., 2009; Sionov et al., 2010; Harrison et al., 2014; Todd et al., 2017; Stone et al., 2019). For example, in *Cryptococcus neoformans*, aneuploidy of chromosomes 1 is associated with resistance to the antifungal drug fluconazole (Sionov et al., 2010) and in *C. albicans* and *C.auris*, chromosome 5 aneuploidy confers fluconazole resistance (Selmecki et al., 2006; Selmecki et al., 2009; Bing et al., 2020). While aneuploidy is an adaptive mechanism for fluconazole resistance in a broad range of fungal pathogens, it has not yet been observed in caspofungin or other echinocandin antifungal drugs. In this study, we found large-scale genome reduction in tetraploid lines and detected smaller-scale increases in genome size in the diploid-evolved lines, indicative of chromosomal aneuploidy across both ploidy states. Of particular importance is copy number variation of chromosome 2 and Chr 5, which are both associated with caspofungin tolerance and identified in 33% (2/6) diploid and 50% (6/12) tetraploid isolates (**Figure 3**).

For some organisms, acquiring resistance to drugs confers a fitness cost in the absence of drug selection (Dahlberg and Chao, 2003; Andersson and Hughes, 2010; Vincent et al., 2013; Hill et al., 2015; Popp et al., 2017). In our study, we found no deficits in the growth rates of caspofungin resistant lines compared to susceptible when grown in the absence of drug (**Figure 5B**), suggesting that caspofungin resistance may not explicitly confer a fitness tradeoff. However, we found that on average, both diploid- and tetraploid evolved lines had growth deficits compared to their ancestral state, in the absence of caspofungin (**Figures 5A**; **S3**). Surprisingly, the magnitude of the growth deficit depended on the initial ploidy state, with diploid-evolved lines exhibiting three-times the deficit of tetraploid-evolved lines. Given the mutagenic nature of caspofungin (Shields et al., 2018; Avramovska and Hickman, 2019), it is likely that caspofungin exposure generated high mutational loads during evolution that are expressed when drug selection is removed. However, since tetraploid-evolved lines undergo genome size reductions within the first 10 days of caspofungin evolution, in which chromosomes are stochastically lost (Hickman et al., 2015), deleterious mutations can be quickly removed from the population. In fact, we see no fitness costs for tetraploid-evolved lines at day 18, following genome size reductions (**Figure 5C**, **TS2**). In contrast, diploid-evolved lines have no clear mechanisms for purging mutations that are deleterious in the absence of selection, and we see significantly slower no-drug growth rates on day 18 compared to the ancestral state (**Figure 5C**, **TS2**).

**Figure 6:**
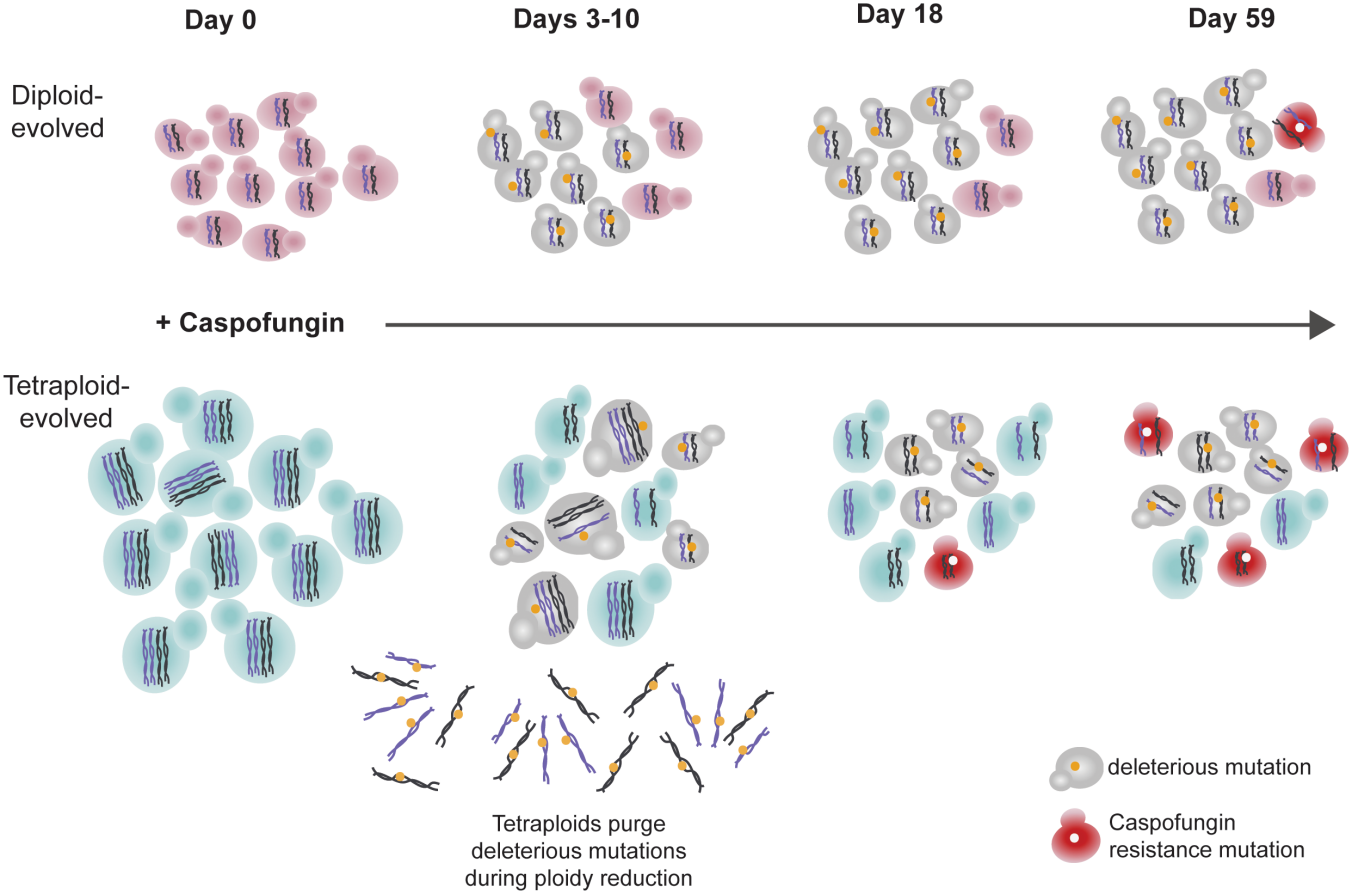
Ploidy reductions purge deleterious mutations and facilitate drug resistance.

In conclusion, we propose that tetraploidy is a transient state with high adaptive potential compared to diploidy (**Figure 6**). This work, along with other experimental evolution studies using yeast species show that asexual whole-genome ploidy transitions occur frequently during short- and long-term evolution (Mable and Otto, 2001; Gerstein et al., 2006; Gerstein et al., 2008; Hickman et al., 2013; Hickman et al., 2015; Selmecki et al., 2015; Gerstein et al., 2017; Turanlı-Yıldız et al., 2017; Harari et al., 2018). These findings, coupled with the observation of ploidy variability within clinical and environmental isolates of various yeast species (Ford et al., 2015; Hirakawa et al., 2015; Wertheimer et al., 2016; Zhu et al., 2016; Ropars et al., 2018; Stone et al., 2019; Gerstein and Sharp, 2021; Scopel et al., 2021) indicate that ploidy transitions may be an important evolutionary force driving microbial eukaryotic adaptation.

## Data availability statement

The data presented in the study are deposited in the Sequence Read Archive (SRA) database, accession number PRJNA892132. The data can be accessed with the following link: https://www.ncbi.nlm.nih.gov/sra/PRJNA892132.

## Author contributions

O.A – Conceptualization, Methodology, Validation, Formal analysis, Writing – Original Draft, Writing – Review & Editing, Visualization, Funding acquisition. ACS – Methodology, Software, Investigation, Data Curation, Formal analysis, Writing – Review & Editing, Visualization. ER – Investigation (Growth Rate data). MAH – Conceptualization, Methodology, Validation, Resources, Writing – Review & Editing, Visualization, Project administration, Funding acquisition, Supervision. All authors contributed to the article and approved the submitted version.

## Funding

This research is supported by NSF DGE-193791 (OA), NIH T32 Training Grant (OA), NSF DEB-1943415 (MH) and Emory University startup funds (MH).

## Acknowledgments

We thank the Emory Flow Cytometry Core, Dr. Fred Winston and the Harvard Curriculum Fellow Programs for their support.

## Conflict of interest

The authors declare that the research was conducted in the absence of any commercial or financial relationships that could be construed as a potential conflict of interest.

## Publisher’s note

All claims expressed in this article are solely those of the authors and do not necessarily represent those of their affiliated organizations, or those of the publisher, the editors and the reviewers. Any product that may be evaluated in this article, or claim that may be made by its manufacturer, is not guaranteed or endorsed by the publisher.
