# Supplementary material for: Tetraploidy accelerates adaptation under drug selection in a fungal pathogen
Source: Front Fungal Biol. 2022 Nov 16;3:984377. doi: 10.3389/ffunb.2022.984377 (PMC10512305; doi:10.3389/ffunb.2022.984377)
Supplement: Supplementary file 1 [file Table_1.docx]

| **Strain** | **Genotype** |  |  |  |  |  |  |  |  |  |  |  |  | |  | | **Reference** | |  |
| --- | --- | --- | --- | --- | --- | --- | --- | --- | --- | --- | --- | --- | --- | --- | --- | --- | --- | --- | --- |
| **Diploids** |  |  |  |  |  |  |  |  |  |  |  |  |  | |  | |  | |  |
| MH84 | *MTLα/α; ura3::imm434::URA3/ura3::imm434; iro1::IRO1/iro1::imm434; his1Δ::hisG/his1Δ::hisG; leu2Δ/leu2Δ; GAL1/gal1Δ::SAT1* | | | | | | | | | | | | |  | |  | | Hickman et.al 2015 | |
| **Tetraploids** |  |  |  |  |  |  |  |  |  |  |  |  |  | |  | |  | |  |
| MH128 | *MTLα/α/a/Δ; ura3Δ/ura3Δ/URA3/URA3, HIS1/HIS1/his1Δ::hisG/his1Δ::hisG; LEU2/LEU2/leu2Δ/leu2Δ; ENO1-GFP:NAT/ENO1/ENO1/ENO1; gal1Δ/gal1Δ/gal1Δ/GAL1;* | | | | | | | | | | | | | | | | | Hickman et.al 2015 | |

**Table S1:** *Candida albicans* strains used in this study.

| Dunn's multiple comparisons test  No-drug growth rate | Summary | Adjusted P Value |
| --- | --- | --- |
| dip0 vs. dip18 | **** | <0.0001 |
| dip0 vs. dip59 | **** | <0.0001 |
| dip0 vs. tet0 | **** | <0.0001 |
| dip0 vs. tet18 | **** | <0.0001 |
| dip0 vs. tet59 | **** | <0.0001 |
| dip18 vs. dip59 | ns | >0.9999 |
| dip18 vs. tet0 | *** | 0.0001 |
| dip18 vs. tet18 | * | 0.0479 |
| dip18 vs. tet59 | ns | >0.9999 |
| dip59 vs. tet0 | **** | <0.0001 |
| dip59 vs. tet18 | ** | 0.0064 |
| dip59 vs. tet59 | ns | >0.9999 |
| tet0 vs. tet18 | ns | >0.9999 |
| tet0 vs. tet59 | ** | 0.0010 |
| tet18 vs. tet59 | ns | 0.1871 |

**Table S2:** Kruskal- Wallis followed by Dunn’s multiple comparison’s test of diploid and tetraploid no-drug growth rates.

**TS2: Statistical summaries for figure 1D – multiple comparisons testing**
