# Supplementary figures and images for: Tetraploidy accelerates adaptation under drug selection in a fungal pathogen
Source: Front Fungal Biol. 2022 Nov 16;3:984377. doi: 10.3389/ffunb.2022.984377 (PMC10512305; doi:10.3389/ffunb.2022.984377)

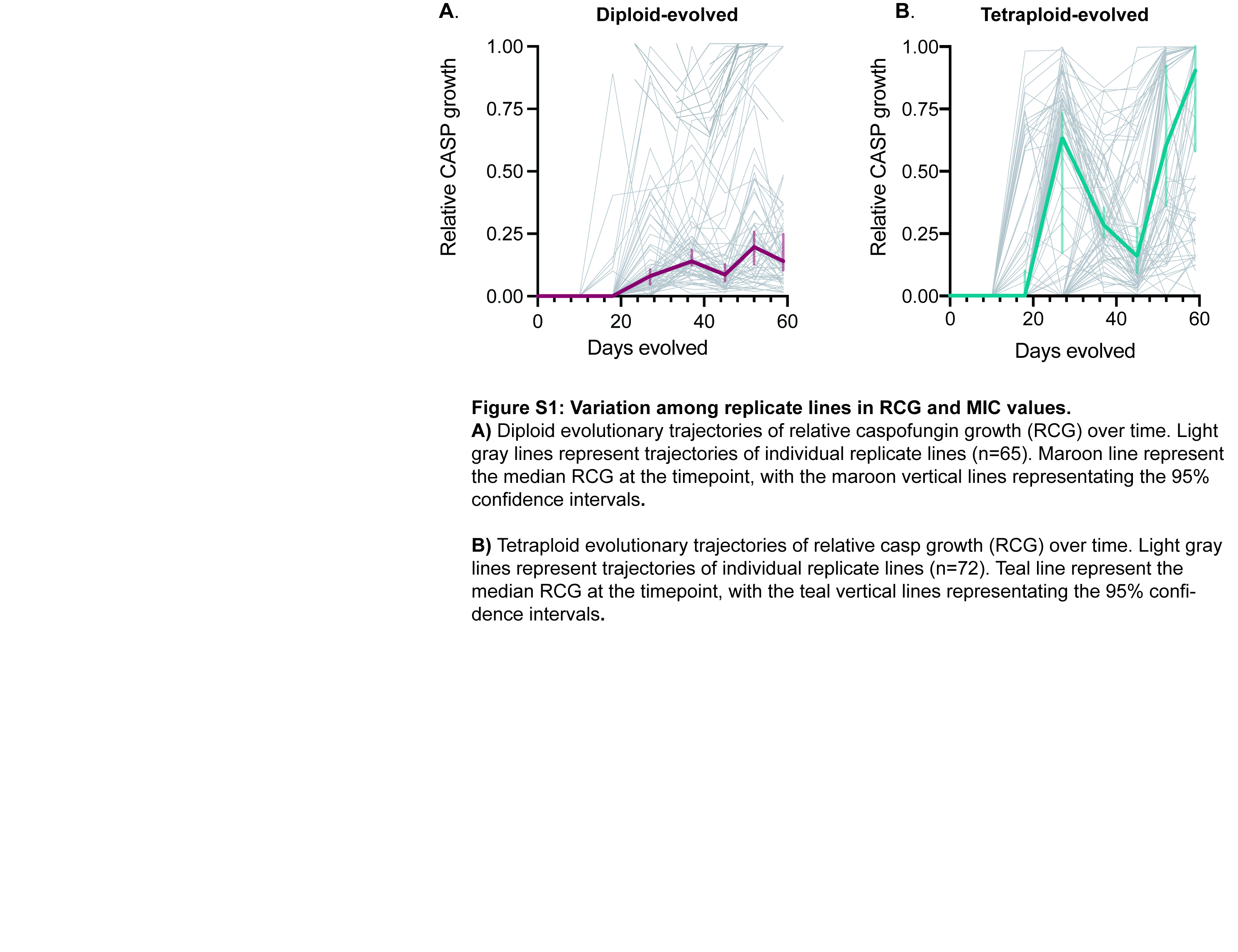

Supplement: Supplementary file 3 [file DataSheet_1.zip › OA Supplemental Figs/2021_07_01 Fig S1.tif]

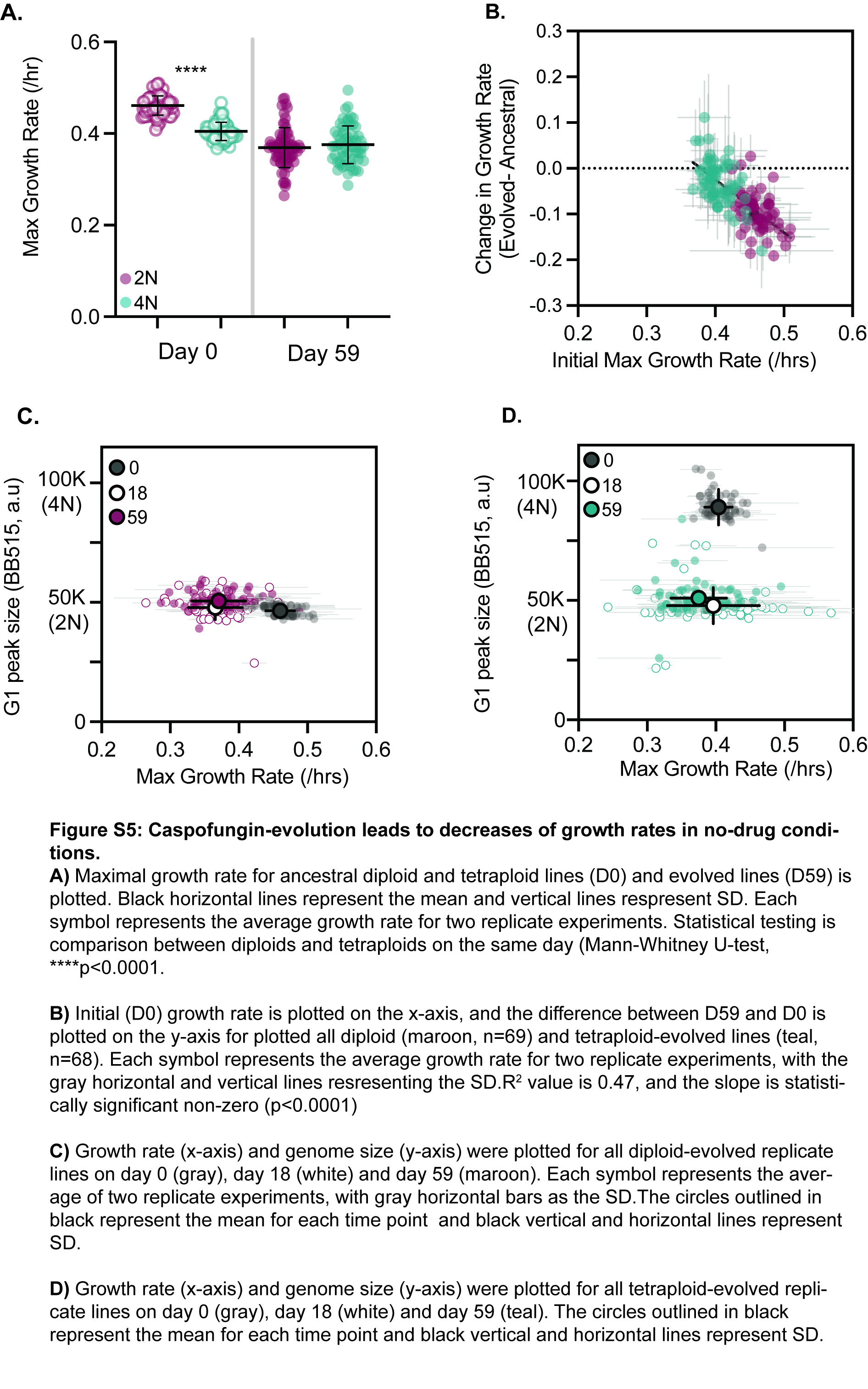

Supplement: Supplementary file 3 [file DataSheet_1.zip › OA Supplemental Figs/2021_07_01 Fig S5.tif]

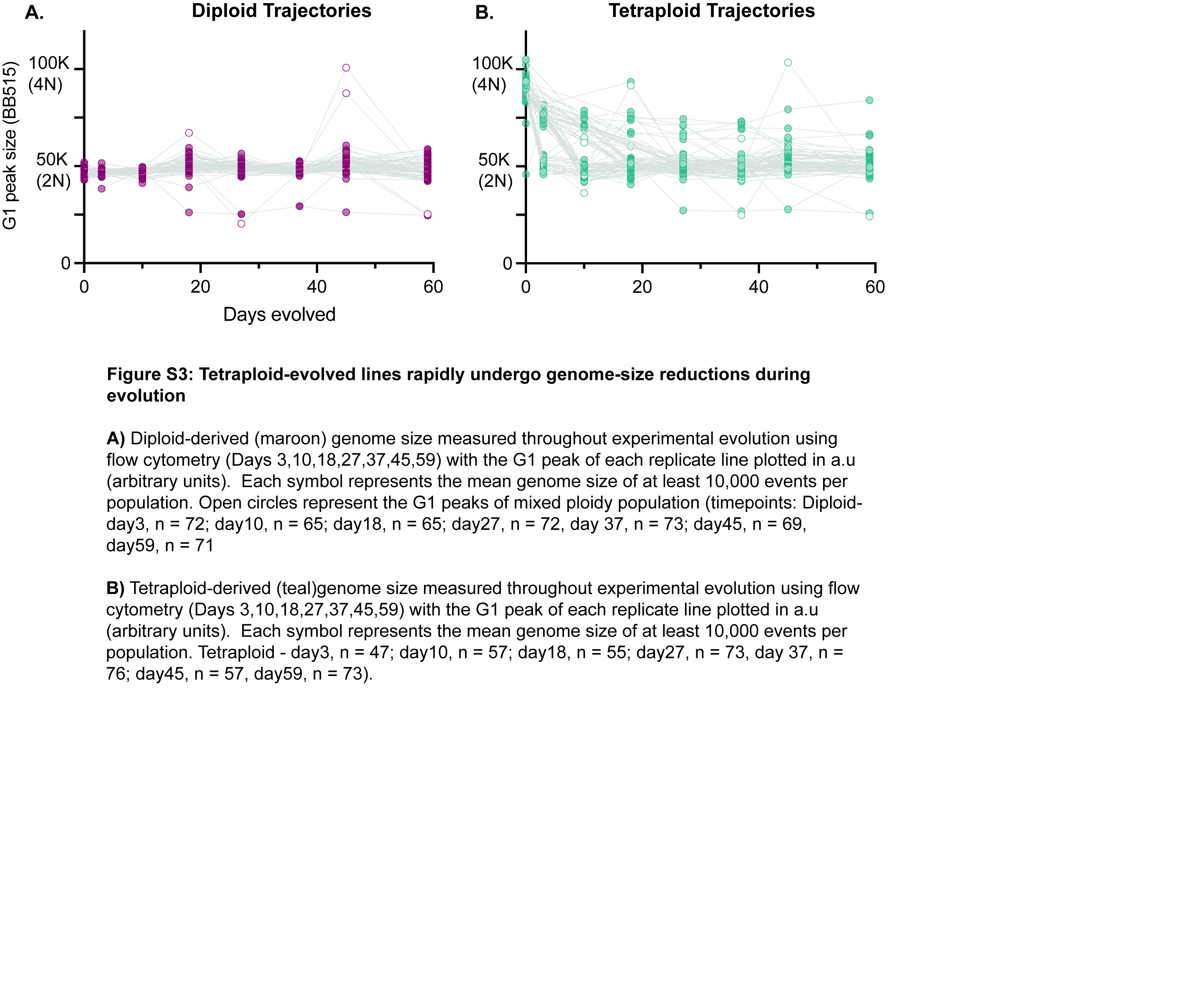

Supplement: Supplementary file 3 [file DataSheet_1.zip › OA Supplemental Figs/2022_07_01 Fig S3 .tif]

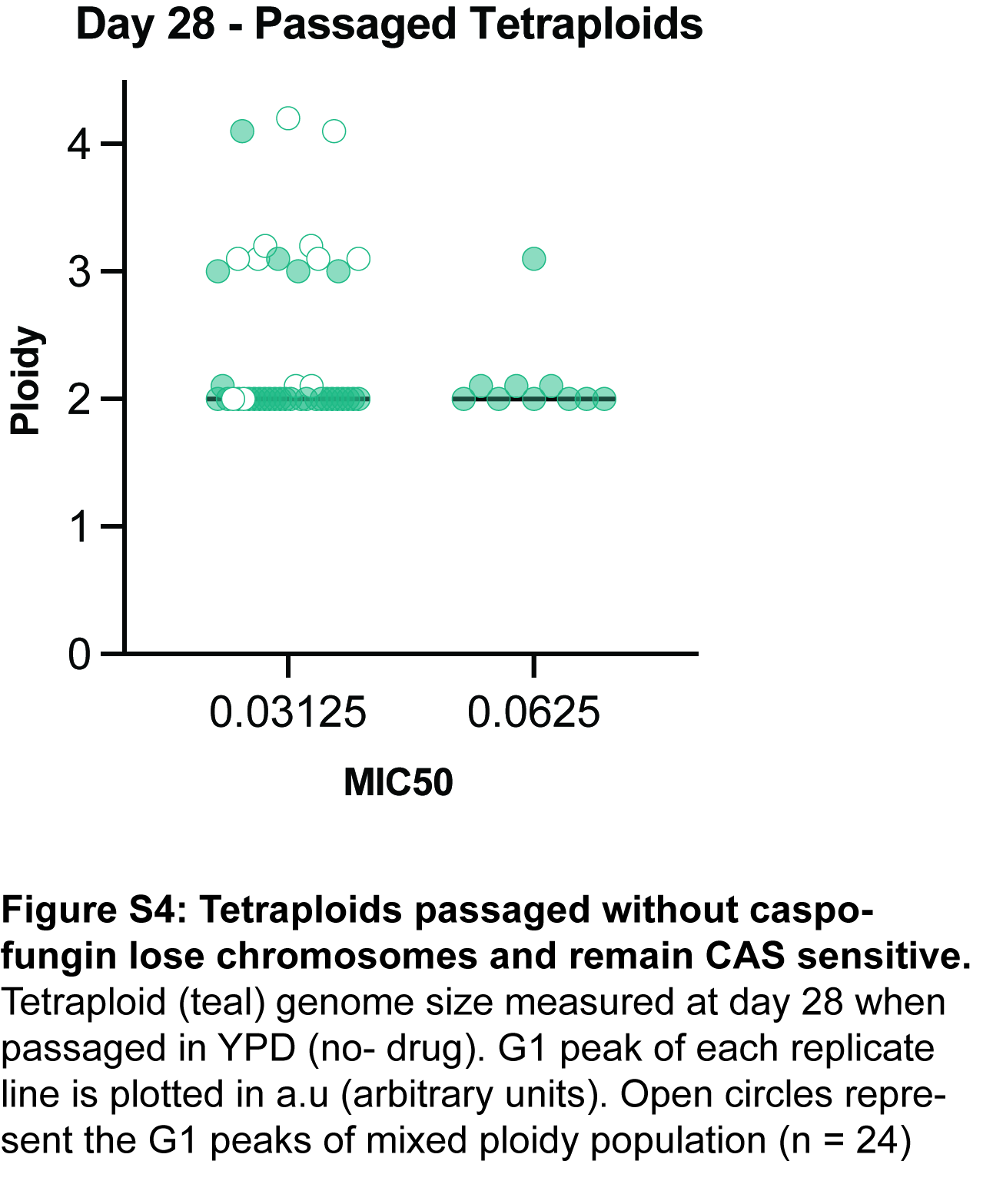

Supplement: Supplementary file 3 [file DataSheet_1.zip › OA Supplemental Figs/2022_07_01 Fig S4 .tif]

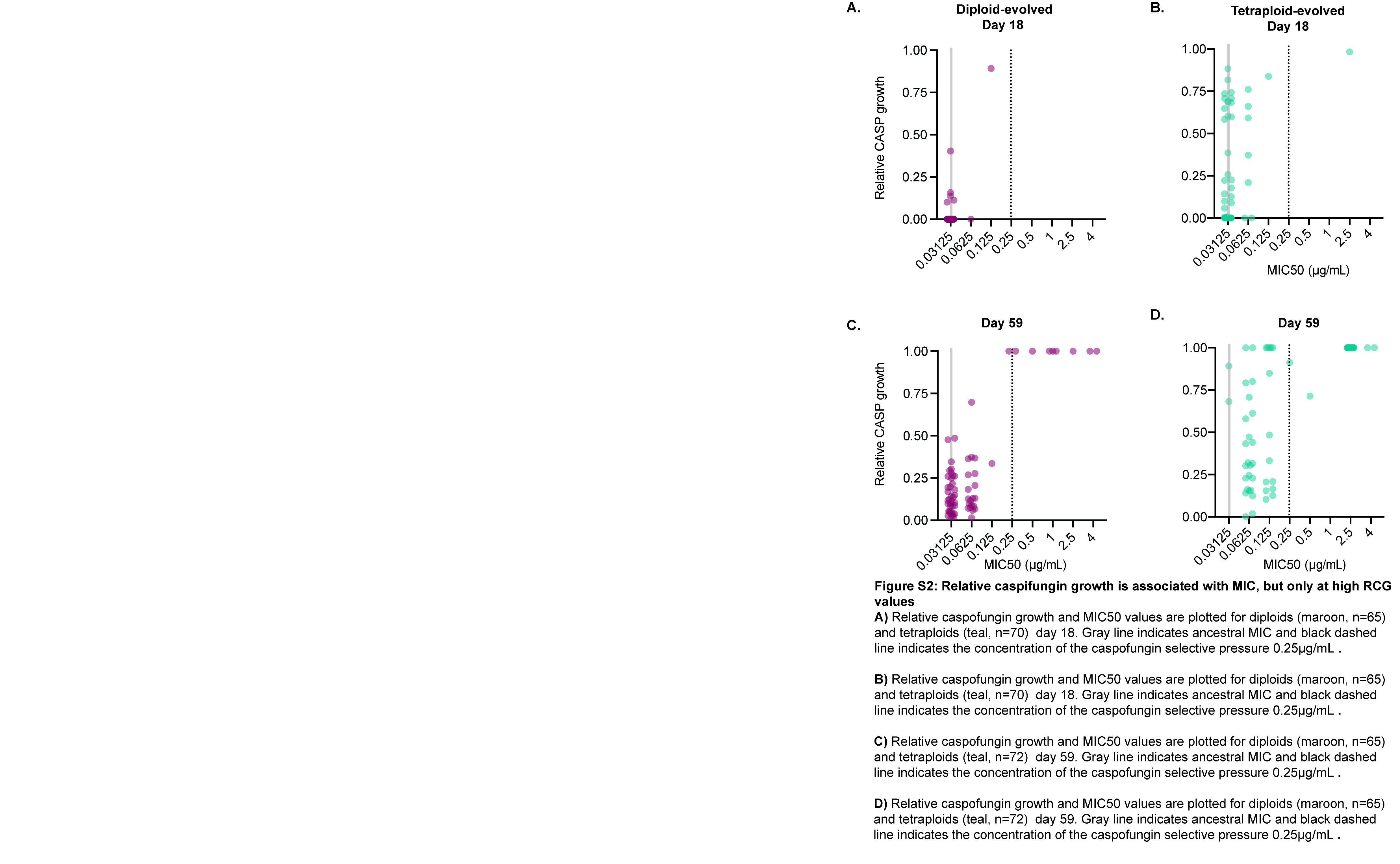

Supplement: Supplementary file 3 [file DataSheet_1.zip › OA Supplemental Figs/2021_07_01 Fig S2 .tif]
